# Recombinant *Lactococcus lactis* Expressing Grass Carp Reovirus VP6 Induces Mucosal Immunity Against Grass Carp Reovirus Infection

**DOI:** 10.3389/fimmu.2022.914010

**Published:** 2022-05-11

**Authors:** Nan Wang, Jiahao Li, Yajun Wang, Yingying Wang, Defeng Zhang, Cunbin Shi, Yingying Li, Sven M. Bergmann, Xubing Mo, Jiyuan Yin, Qing Wang

**Affiliations:** ^1^Key Laboratory of Fishery Drug Development of Ministry of Agriculture, Key Laboratory of Aquatic Animal Immune Technology of Guangdong Province, Pearl River Fisheries Research Institute, Chinese Academy of Fishery Sciences, Guangzhou, China; ^2^Institute of Infectology, Friedrich-Loffler-Institut (FLI), Federal Research Institute for Animal Health, Greifswald–Insel Riems, Germany

**Keywords:** grass carp reovirus, *Lactococcus lactis*, oral vaccine, VP6, mucosal immune protection

## Abstract

Grass carp haemorrhagic disease caused by grass carp reovirus II is a serious disease of the aquaculture industry and vaccination is the only effective method of GCRV protection. In this study, *Lactococcus lactis* was used as oral vaccine delivery to express the GCRV II VP6 protein. We evaluated the protective efficacy of the live vaccine strain to induce mucosal immune protection. After oral administration, the recombinant strains remained in the hindgut for antigen presentation and increased the survival rate 46.7% and the relative percent survival 42.9%, respectively versus control vaccination. Though *L. lactis* alone can induce the inflammatory response by stimulating the mucosal immune system, the recombinant *L. lactis* expressing VP6 greatly enhanced nonspecific immune responses *via* expression of immune related genes of the fish. Furthermore, both systemic and mucosal immunity was elicited following oral immunization with the recombinant strain and this strain also elicited an inflammatory response and cellular immunity to enhance the protective effect. *L. lactis* can therefore be utilized as a mucosal immune vector to trigger high levels of immune protection in fish at both the systemic and mucosal levels. *L. lactis* is a promising candidate for oral vaccine delivery.

## Introduction

Outbreaks of grass carp haemorrhagic disease (GCHD) caused by grass carp reovirus (GCRV), result in mass mortality of grass carp and serious economic losses for the aquaculture industry (1). GCRV can be classified into three different genotypes, and the results of epidemiological investigation showed that GCRV II is the major epidemic strain. An injectable vaccine has proven effective against GCRV II infections but is not an ideal immunization route due to its complex operations (2, 3). Oral immunization has many advantages such as easy operation and low labor input and can be applied to different culture conditions (4). However, environmental exposure of oral vaccines has led to antigen degradation that impeded the potency of oral vaccines (5).

*Lactococcus lactis* is a species of lactic acid bacteria and is a recognized safety grade microbe that has been used in oral immunization trials (6–8). *L. lactis* is Gram-positive bacterium and is therefore free of LPS (endotoxin) and can survive the intestinal tract and has been used as a tool for antigen presentation (9–11). Furthermore, *L. lactis* has multiple probiotic functions such as gut microecology regulation, improving feed utilization efficiency and enhancing innate and acquired immunity (12–14). These advantages of *L. lactis* may therefore allow its use as a carrier to express foreign antigenic proteins.

In order to develop an appropriate oral subunit vaccine, antigen choice is as important as the selection of a suitable delivery system (15). As with all reoviruses, GCRV II is organized as two concentric icosahedral layers: an inner core and an outer capsid layer (16). VP6 is one of the primary structural protein components of the GCRV II mature particle and is encoded by segment 9 and forms part of the viral core and plays an important role in replication (17). In particular, VP6 functions as a mediator that bridges the inner core and outer capsid and is directly inserted into position co-translationally without the involvement of ATP or chaperones. VP6 can directly bind target cell surface sialic acids (18). The VP6 protein has been shown to be immunogenic and elicit the production of neutralizing antibodies (19). Therefore, VP6 is a potential candidate protein for the development of a GCRV II oral vaccine.

GCHD is not confined to grass carp, and the rare minnow (*Gobiocypris rarus*) is as sensitive to GCRV II as grass carp (20). In addition, the use of the rare minnow as the model for evaluation of experimental vaccines against GCRV II infections has many advantages such as its small size, facile care, transparent egg membrane and annual spawning. These conditions are ideal for a model experimental aquatic animal. In addition, the immunogenicity and protective effects of vaccines for grass carp and rare minnow are consistent in their expression of immune-related genes, specific serum antibody titers and protection (21).

In this study, we constructed a recombinant *L. lactis* displaying the VP6 protein and administered the recombinant strain to rare minnow cultures that were then challenged with GCRV II. The goal of this work was to investigate whether *L. lactis* expression of VP6 would stimulate mucosal immunity and protection.

## Materials and Methods

### Experimental Fish

Rare minnows approximately 4.0 ± 0.5 cm in length were obtained from the Institute of Hydrobiology, Chinese Academy of Sciences (Wuhan, China) and acclimatized in the laboratory for 2 weeks before experimental manipulations. Fish were fed a commercial diet daily. Water temperature was maintained at 28°C. Prior to experiments, fish were randomly selected and liver, kidney and spleens were examined for the presence of GCRV using RT-qPCR as described previously (21). All animal procedures were conducted according to animal welfare standards and approved by the Ethical Committee for Animal Experiments of Pearl River Fisheries Research Institute, Chinese Academy of Fishery Sciences, China. All animal experiments complied with the guidelines of the Animal Welfare Council of China.

### Strains

The GCRV II (GCRV HuNan1307 strain) and *L. lactis* NZ9000 were kept in our laboratory. GCRV was propagated in cultured grass carp swim bladder cells at 28°C in M199 medium containing 5% fetal bovine serum. *L. lactis* NZ9000 was cultured at 30°C in M17 broth medium without shaking. LD_50_ determinations were performed according to the method of Reed-Muench (22). Viral titers were determined by the half lethal dose (LD_50_) method and was typically 10^–5.25^ LD_50_/20 μL for the rare minnow.

### Bioinformatics Analysis the Structure of VP6

To determine a suitable segment of VP6 expressed by *L. lactis* NZ9000, a protein structure set was obtained from the SWISS-MODEL web server. The transmembrane area of VP6 was analyzed with TMHMM 2.0, the secondary structure was analyzed by the PSIpred-MEMSAT 3 web server and the functional domains was analyzed through the online software the PredictProtein server.

### Construction of pNZ8148-*VP6* Recombinant Plasmid

The designed gene construct was amplified by PCR using the primers GCRV VP6-F/R (**Table 1**). Amplicons were subcloned into plasmid pNZ8148 (Novagen, Madison, WI, USA) using *Nco* I and *Xba* I restriction enzymes resulting in recombinant pNZ8148-*VP6*. The recombinant plasmid was introduced into *L. lactis* NZ9000 by electroporation as previously described (23). The *L. lactis* transformants were cultured in M17 agar containing 5 μg/mL chloramphenicol and incubated at 30°C for 24 h. Positive clones were selected and confirmed by PCR using VP6 primers (**Table 1**).

**Table 1:** Primers used in this study.

Primer name	Sequence (5′–3′)
GCRV VP6	F: 5'-CCATGGGTAAGACATTCGACGTCGGGACG-3' R: 5'-TCTAGAATGGTGATGGTGATGATGCGTCAAGCTTGTCAACACGCTGCTCCC-3'
β-actin	F: 5'-CTATGTTGGTGACGAGGCTCA-3' R: 5'-CCCAGTTGGTGACAATACCG-3'
TLR3	F: 5'-TTGGTAGAGGCTAATGCG-3' R: 5'-AATGGAGGACAACCGAGA-3'
TLR5	F: 5'-AAGGGTGCTTGGAGATAA-3' R: 5'-TTGAAAGTCCCAGATGAA-3'
MyD88	F: 5'-GGTGGTAATTTCCGATGA-3' R: 5'-GTAGACAACAGGGATAAGG-3'
NF-κB	F: 5'-AACTCAGTCAGGCTCCATTGC-3' R: 5'-GACAGTGCTCTCCGTCTTTCC-3'
IFN2	F: 5'-ACAGTCAAGCAGGAGGAGGA-3' R: 5'-TCACTGGCGCTGTCTGTATC-3'
Mx	F: 5'-GACACGCTGTCCTCTGGTAT-3' R: 5'-CAGTTTCTTTGTTTGGCTCTG-3'
IRF7	F: 5'-CCAAGAGCAGAGCCAGTT-3' R: 5'-TAGGGCGTCCCAAAGTAG-3'
MHC II	F: 5'-AATGACGACGGCACTTACAA-3' R: 5'-ACTCCCAGCAGCCCCAGA-3'
IL-1β	F: 5'-TGATGAGATGGACTGCCCTG-3' R: 5'-TGTCCGTCTCTCAGCGTCAC-3'

Underlined were the restriction site.

### Expression and Identification of the VP6 Protein in *L. lactis*

The recombinant strain was cultured in M17 broth medium containing 10 μg/mL chloramphenicol 30 °C until log growth phase. Protein expression was induced by the addition of using nisin (Weijia, Guangzhou, China) for 4 h. *L. lactis* cells were then collected by centrifugation and the pellets were suspended in PBS. The cells were lysed with the addition of 10 mg lysozyme and incubation at 37°C for 1 h. The cell lysates were then further processed as described previously (23). Protein production was analyzed using 12% SDS-polyacrylamide gel electrophoresis (SDS-PAGE) and electrotransferred to polyvinylidene fluoride membranes (Millipore, Billerica, MA, USA). The membranes were then incubated with anti-rVP6 polyclonal antibody (24) at 1:1000 in PBS-Tween-20 (PBST) for 2 h at 37°C and followed by incubation with the secondary antibody HRP-conjugated goat anti-rabbit IgG (1:5000 diluted with PBS (Sigma-Aldrich, Pittsburg, PA, USA) and visualized with the addition of diaminobenzidine (Sigma-Aldrich).

Aliquots of protein (8 μg/mL) were added to 96-well plates and incubated at 4°C overnight and washed 3× with PBST and blocked with 5% skim milk in PBS for 2 h at 37°C. Anti-rVP6 polyclonal antibody (24) was then added at 1/1000 dilution in PBS for 2 h at 37°C and washed 3× with PBST and then incubated with HRP-conjugated goat anti-rabbit IgG (1:5000 Sigma-Aldrich) for 1 h at 37°C. OPD-H_2_O_2_ substrate (Beyotime, Canton, China) was then added and the plates were incubated for 10 min. The reaction was stopped by addition of 2M H_2_SO_4_ and absorbance was measured in a microplate reader (Biotek, Santa Clara, CA, USA) at 450 nm. Samples were assayed in triplicate.

### Oral Immunization and Challenge

Immunization trials were conducted on specific pathogen-free rare minnows (100 fish per group). On 1, 2, 3, 21, 22 and 23 days, the fish were orally immunized with 10 μL 2 × 10^9^ CFU/mL/fish/d of the recombinant strain and control fish were exposed to with *L. lactis* NZ9000 or PBS as controls (25, 26). At 3-, 7-, 14- and 21-days post-vaccination (dpv), spleen, kidney and hindgut tissues were dissected for RNA extraction and stored at -80°C. During the immunization procedure, the fish were bled twice at weeks 3 and 6 using 10 fish per group. Samples were obtained by tail ablation and collected using heparinized microcapillaries and kept at -80°C for ELISA. At 42 dpv 30 fish from vaccinated and control groups were challenged by injection with GCRV HuNan1307. Fish mortality was monitored daily for 14 days and fish tissues were examined using RT-qPCR to quantify the viral loads using the newly established method in our laboratory. The relative percentage survival (RPS) (27) was calculated by the following formula: RPS = [1-(% mortality of vaccinated fish/% mortality of control fish)] × 100.

### Detection of Recombinant Strain Colonization in Intestines

After oral immunization with 10 μL 2×10^9^ CFU/mL/fish, the hindgut tissues were recovered from sacrificed fish at 3, 6, 12, 24, 48 and 72 h after the first immunization and homogenized in PBS before plating serial dilutions on M17 agar plates containing chloramphenicol (5 μg/mL). Several clones were picked up randomly and analyzed using PCR amplification to detect the presence of the VP6 gene (see above).

### Determination of Immune-Related Gene Expression by RT-qPCR

RT-qPCR was used for relative quantitative analysis of the rare minnow cDNA obtained from spleen, kidney and hind gut tissues. The immune-related genes included Toll-like receptors 5 (TLR5), 3 (TLR3), myeloid differentiation primary response protein 88 (MyD88), nuclear factor kappa beta (NF-kB), interferon regulatory factor 7 (IRF7), Myxovirus resistance factor (Mx), interferon 2 (IFN2), major histocompatibility complex class II (MHC II) and interleukin-1β (IL-1β). β-actin was used as a reference gene for normalization (**Table 1**). RT-qPCR was performed using a SYBR Premix Ex Taq kit (Takara, Shiga, Japan) on an ABI 7500 fluorescence quantifier (Applied Biosystems, Foster City, CA, USA) with the following procedures: 95°C for 5 min and 35 cycles of 95°C for 15 s, 60°C for 45 s in triplicate. The expression level of immune genes was calculated using the 2^-△△Ct^ method (21). All data were expressed as the mean ± SD.

### Detection of Specific Antibody Levels by ELISA

To test the specific IgM levels against VP6 in the antisera of immunized fish, anti-IgM MAbs were prepared and kept in our laboratory. Serum antibody levels were determined by ELISA as described previously (23). Briefly, recombinant VP6 (rVP6) (24) (8 μg/mL in carbonate-bicarbonate buffer) was used as antigen to coat wells overnight at 4°C. After blocking with 5% skim milk at 37°C for 2 h, serum samples (1:50) were added and the plates were incubated for 2 h at 37°C and washed 3× with PBST and then incubated with rabbit anti-grass carp IgM (21) (1:1000) for 1 h at 37°C followed by incubation with HRP-conjugated goat anti-rabbit IgG (1:5000) for 1 h at 37°C. The wells were developed by reaction with tetramethylbenzidine substrate (Beyotime) for 10 min and the reaction was stopped by addition of 2 M H_2_SO_4_. OD values at 450 nm were taken as described above. Results of 10 samples were averaged to obtain the antibody titers of immunized fish at the different sampling times.

### Statistical Analysis

The experimental data was expressed as mean ± SD and the P values were calculated using one-way ANOVA with a Dunnett *post-hoc* test (SPSS Statistics, version 22.0, IBM, Chicago, Ill, USA). A P value of < 0.05 was considered significant, and < 0.01 was greatly significant.

## Results

### Identification of the Recombinant Strains

**Figure 1:**
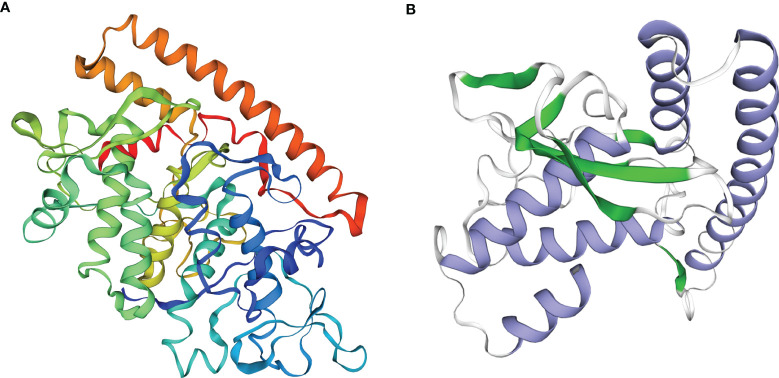
Function and structure predicted of the GCRV VP6. **(A)** Analysis of functional domains of the GCRV VP6. **(B)** Structure prediction of the N-terminal of the GCRV VP6.

**Figure 2:**
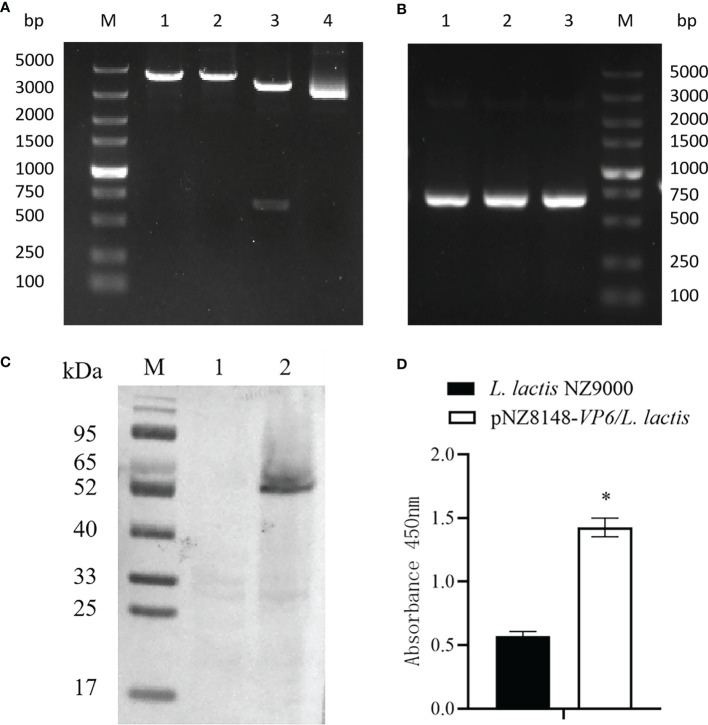
Identification of the recombinant strain pNZ8148-*VP6*/*L. lactis*. **(A)** Identification by enzyme digestion. M: DNA maker DL5000; Lane 1: *Nco* I digestion; Lane 2: *Xba* I digestion; Lane 3: double digestion; Lane 4: pNZ8148-*VP6* plasmid. **(B)** Identification of recombinant plasmids pNZ8148-*VP6* by PCR. Lane M: DNA marker DL5000, Lanes 1–3: *L. lactis* pNZ8148-*VP6*. **(C)** Western blot analysis of pNZ8148-*VP6* fusion protein expression, M: Low molecular weight protein maker. Lane 1: negative control *L. lactis* NZ9000. Lane 2: *L. lactis* pNZ8148-*VP6*. **(D)** Indirect ELISA analysis of pNZ8148-*VP6* fusion protein expression. *p < 0.05.

The VP6 protein contained two functional domains; an N-terminal fibrous tail arranged as a triple coiled coil that serves as a virion anchor and a C-terminal globular head that interacts with the cellular receptor. These two parts form by separate trimerization events. The N-terminal fibrous tail forms on the polysome and post-translational assembly of the C-terminal globular head requires Hsp90 and binds target cell surface sialic acids and may promote apoptosis (**Figure 1A**). We examined the predicted structure of the N-terminus and identified 5 α-helices (residues 578-588, 596-613, 670-685, 721-737 and 740-775) connected by short aperiodic coils. The N-terminal of VP6 possessed no other complexities and was therefore suitable for the prokaryotic expression system (**Figure 1B**). GCRV II *VP6* was amplified by PCR and cloned into the expression vector pNZ8148 and the recombinant plasmid was verified using double digestion (**Figure 2A**) and PCR **(** **Figure 2B**). Western blots following expression in *L. lactis* indicated the presence of 57.6 kDa bands from recombinant strains that were lacking in vector only controls (**Figure 2C**). In addition, indirect ELISA indicated a significant (p < 0.05) increase in the presence of the recombinant protein versus time of exposure to the inducer nisin (**Figure 2D**).

### Intestinal Colonization

**Figure 3:**
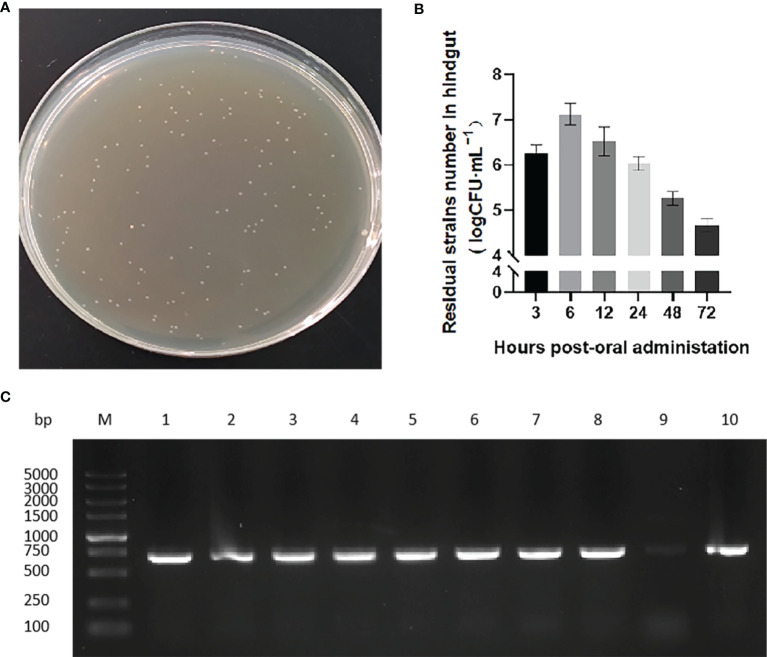
Colonization of strains in rare minnow intestinal tract. **(A)** Recombinant strain colonies in pNZ8148-*VP6* group that grew on chloramphenicol M17 agar plates. **(B)** Colony counts obtained from hindgut tissues collected at the indicated time points following immunization. The data represent means ± SEM of 3 fish. **(C)** PCR specific amplification the fragment of pNZ8148-*VP6* in colonies from *L. lactis* pNZ8148-*VP6* group intestinal dilution contents. Lane M: DNA marker DL5000, Lanes 1–9: colonies picked from plates.

We next evaluated the transit of pNZ8148-*VP6/L. lactis* in the rare minnow intestine. After exposure to the recombinant bacteria strain, hindgut samples at 3, 6, 12, 24, 48 and 72 h following the first immunization were collected and isolated chloramphenicol-resistant bacterial clones immediately (**Figure 3A**). The numbers of these colonies increased over time and reached a maximum at 6 h. The colony numbers then decreased but could still be detected and persisted at 72 h (**Figure 3B**). Isolated bacterial colonies also possessed the VP6 gene (**Figure 3C**) indicating the presence of the recombinant *L. lactis* in the intestines of immunized fish.

### Immunoprotective Effects of the Vaccine

**Figure 4:**
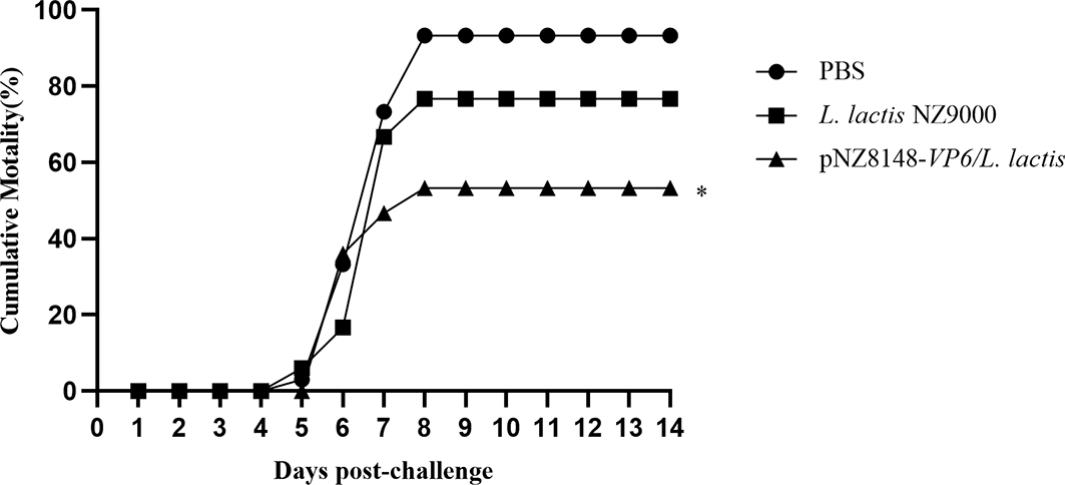
Cumulative mortality curves of vaccinated fish exposed to GCRV-HuNan1307. Mortality was monitored daily for 14d post-challenge. *P < 0.05 between immunized and control groups at day 14.

Immunized fish were challenged with 10 LD_50_ of GCRV HuNan1307 at 42 dpv and monitored for mortality and clinical symptoms for 14 d. All moribund fish presented typical clinical symptoms of GCHD including a deepening of body color, unresponsiveness and organ hemorrhaging. GCRV II could be detected in the tissues of all moribund fish. Fish mortality was first observed at 5 days post-infection in the PBS and *L. lactis* NZ9000 groups but 1 day later in the pNZ8148-*VP6/L.lactis* group. Mortality peaked for each group on day 8 following challenge. The cumulative mortality rate for the pNZ8148-*VP6/L. lactis* group was 53.3% and significantly (P<0.01) lower than the cumulative mortality for the PBS (93.3%) and the *L. lactis* NZ9000 (76.7%) groups. Hence, the protective efficacy of pNZ8148-*VP6/L. lactis*, compared with the PBS control yielded an RPS of 42.9%. These data indicated that the recombinant *L. lactis* greatly increased the survival of immunized fish against GCRV II infection (**Figure 4**).

### Detection of Specific Antibody Levels in Serum

**Figure 5:**
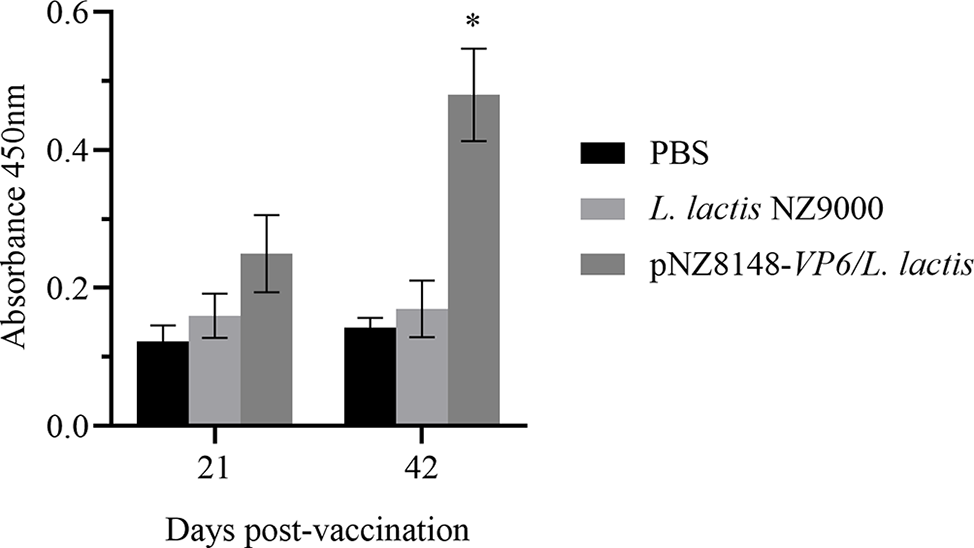
Detection of serum specific antibody levels by ELISA. Levels are indicated using OD450 values and presented as mean ± SD. A comparison the *L. lactis* pNZ8148-*VP6* group and the PBS group is shown. *p < 0.05.

The protective effects of the recombinant vaccine strains were then examined for their ability to induce immunity by measuring levels of specific anti-VP6 antibodies in the sera of immunized fish. Anti-VP6 levels were significantly elevated in the fish receiving the specific vaccine strain 21 days after the booster vaccination (p<0.05) and were even higher 42 days later (p<0.05). There were no significant differences in the level of specific serum antibody for the controls (**Figure 5**). These results indicated that the VP6 protein could be successfully delivered to the mucosal immune system of the fish by *L. lactis* and efficaciously induced the systemic immune response in the rare minnows.

### Expression of Immune-Related Genes in Different Tissues

**Figure 6:**
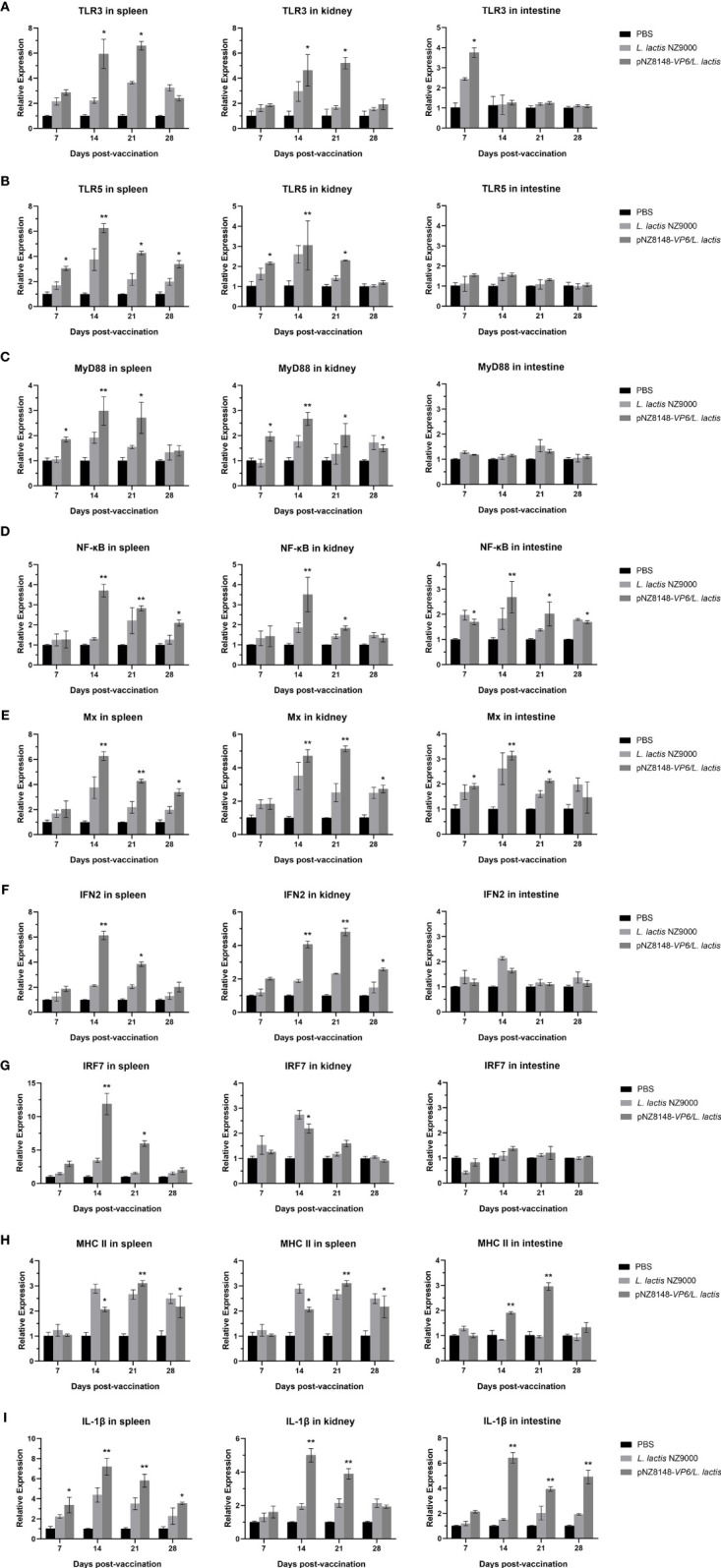
RT-qPCR analysis of the expression of immune-related genes in different tissues of the rare minnow. **(A)** TLR3, **(B)** TLR5, **(C)** MyD88, **(D)** NF-κB, **(E)** Mx, **(F)** IFN2, **(G)** IRF7, **(H)** MHC II, **(I)** IL-1β. The mRNA level of each gene was normalized on the basis of β-actin gene expression. *p < 0.05, **p < 0.01 (versus PBS group).

Immune-related gene expression was then used to evaluate the immunized and control fish using RT-qPCR of cDNA derived from intestinal, spleen and kidney tissues. The relative expression levels of all the genes we used for screening with the exception of actin were significantly or greatly significantly up-regulated in the pNZ8148-*VP6/L. lactis* immunization group. This indicated that the oral immune recombinant strain induced both cellular and humoral immune responses in the fish. Specifically, intestinal levels of TLR3 were significantly up-regulated 7 dpv (p<0.05) and no significant changes in TLR5 expression were observed (p>0.05) (**Figures 6A, B**). Furthermore, although TLR3 expression was consistently increased 14 dpv in spleen and kidney (p<0.05), in the intestine it returned rapidly to basal levels. In the intestine, the expression level of genes downstream of TLR3 and TLR5 including NF-κB, IFN2 and Mx were also up-regulated significantly as in the spleen and kidney. In contrast, MyD88 and IRF7 in the intestine were not significantly altered (**Figures 6C–G**). The expression levels of MHCII in spleen, kidney and intestine were significantly increased on days 14 and 21 and returned to baseline levels at day 28 (**Figure 6H**). These results suggested that the pNZ8148*-VP6/L. lactis* strain could induce adaptive cellular immunity in both the mucosal and general immune systems. Additionally, the up-regulation of IL-1β expression could be observed in spleen, kidney and hind gut in the pNZ8148*-VP6/L. lactis* group indicating that the vaccine strain could cause an inflammatory response in the fish after oral immunization (**Figure 6I**).

## Discussion

Vaccination is considered the preferred method for reducing the severity of GCHD. Although attenuated vaccines are efficacious, they are limited because they must be physically injected in each fish and this is not compatible with the large sample sizes used in the fish culture industry. Oral vaccines are more convenient for farmers (28–31). In this study, we constructed a recombinant *L. lactis* strain that displayed the VP6 protein and exposure to this strain could effectively induce immune protection in the fish *via* oral administration.

In previous studies, VP4 (27), VP35 (28) and NS38 (29) have been tested as oral vaccine candidates. Although all these subunit vaccines generated certain levels of immune protection, the effectiveness was not as good as the injected live vaccine. The immunoprotective effects of these antigens are related to the robustness of the antigen. VP6 is a capsid component and our bioinformatics analysis indicated that the fibrous tail anchor also possessed a small region which could bind target cell surface sialic acids and may promote apoptosis. This region could elicit neutralizing antibodies and the structure was simple enough to be expressed in the prokaryote *L. lactis.*

Probiotics have been previously used as live vector vaccine systems and can be an effective antigen delivery system (32). *L. lactis* can symbiose in the gut and is safe for aquaculture and terrestrial animals to promote intestinal health (33–35). In this study, *L. lactis* was used to deliver the oral vaccine displaying the N-terminus of VP6. The recombinant strain could be continuously isolated and detected in the intestinal tract over 72 h indicating a compatibility with the fish gut. This also resulted in stimulation of mucosal immunity. These results were consistent with the uses of *L. lactis* in colonization of the intestinal tracts of terrestrial animals (26).

In order to explore the mucosal immune effect and mechanism for the generation of immunity with the recombinant *L. lactis*, the rare minnow was used as the model for evaluation of the oral vaccine. We assessed the cumulative mortality rate of vaccinated fish challenged with GCRV. Compared with the naive (PBS) group, the survival rate and the relative percent survival were 46.7% and 42.9%, respectively. Additionally, compared with the oral vaccines using *Bacillus subtilis* as the delivery system, the *L. lactis* presented better immunoprotective effects in fish (26). The same antigen and different inoculation routes and methods may lead a different effects for different immune activation pathways (28, 36) and immunoprotection of VP6 by injection was better than by the oral methods (37). The focus of future research will be the choosing of a proper immunopotentiator to improve the immune protection effect.

Our *L. lactis* vaccine generated specific IgM production in serum that was significantly (p<0.05) elevated over controls. IgM is the most important Ig isotype for teleosts and the most abundant immunoglobulin in blood and in mucosal-associated lymphoid tissue (MALT). IgM therefore plays a key role in mucosal immunity (38). Our results indicated that oral pNZ8148-*VP6*/*L. lactis* could effectively induced systemic humoral immune response in the fish.

Recombinant probiotic vaccine strains may also generate similar immunoprotective effects through different mechanisms. Oral administration of antigens can elicit an effective immune response by regulation of cytokines or other factors (39). For instance, MHC II levels on the surface of antigen-presenting cells (APC) are rapidly and significantly up-regulated in spleen, kidney and intestine. Our results indicated that the antigen carried by *L. lactis* was also taken up by professional APCs in the gut. These APCs can interact directly with CD4+ T helper cells to stimulate fish immunity (40–42).

The innate immunity of fish is also activated *via* pattern recognition receptors (PRR) (43) that are responsible for sensing the presence of pathogen-associated molecular patterns. TLR3 and TLR5 as the classical PRRs and are important sensors for the presence of invading microorganisms and play important roles in antiviral immunity (44, 45) These function *via* MyD88-dependent and MyD88-independent signaling pathways (46). We found that TLR3 expression first increased in intestinal tissues and 7 days later was increased in the spleen and kidney. TLR5 and MyD88 were up-regulated in the spleen and kidney 14 dpv. These results indicated that the APCs have the ability to directly activate innate immune signaling pathways in the immune tissue of the intestine. The recombinant vaccine strain also induced expression of inflammatory and cytokine related genes such as NF-κB, Mx, IRF7 and IFN2. Elevation of NF-κB was followed by Mx in spleen and kidney as well as the intestine. Additionally, vaccination induced a large-scale amplification of the signaling cascade that activates IFNs *via* IFN regulatory factors in the spleen and kidney. Although the expression level of IRF7 in the intestine was not up-regulated, some of the downstream genes including IFN2 were up-regulated. This was most likely a consequence of compensatory regulation from the spleen or kidney.

In summary, we constructed a recombinant *L. lactis* vaccine strain that displayed the GCRV VP6 protein. After oral immunization, the strain could reside in the hindgut of the fish at least 72 h to induce mucosal as well as systemic immunity. Fish survival of a GCRV challenge was also significantly enhanced by vaccination. This work highlights the use of *L. lactis* as a powerful oral vaccine delivery system in aquatic systems and may be used for other oral vaccines.

## Data Availability Statement

The original contributions presented in the study are included in the article/supplementary material. Further inquiries can be directed to the corresponding authors.

## Ethics Statement

The animal study was reviewed and approved by the Ethical Committee for Animal Experiments of Pearl River Fisheries Research Institute, Chinese Academy of Fishery Sciences, China. All animal experiments complied with the guidelines of the Animal Welfare Council of China.

## Author Contributions

All authors listed have made a substantial, direct, and intellectual contribution to the work and approved it for publication.

## Funding

This work was supported by National Key R&D Program of China [grant numbers 2019YFD0900103], the China Agriculture Research System of MOF and MARA [grant numbers CARS-45], Central Public-interest Scientific Institution Basal Research Fund (CAFS 2020TD45), Central Central Public-interest Scientific Institution Basal Research Fund, CAFS [grant number 2021SJ-XK1], Guangdong Provincial Special Fund For Modern Agriculture Industry Technology Innovation Teams [grant numbers 2021 KJ150], and National Freshwater Genetic Resource Center [grant number FGRC-18537].

## Conflict of Interest

The authors declare that the research was conducted in the absence of any commercial or financial relationships that could be construed as a potential conflict of interest.

## Publisher’s Note

All claims expressed in this article are solely those of the authors and do not necessarily represent those of their affiliated organizations, or those of the publisher, the editors and the reviewers. Any product that may be evaluated in this article, or claim that may be made by its manufacturer, is not guaranteed or endorsed by the publisher.
